# Identification of *TUBB8* Variants in 5 Primary Infertile Women with Multiple Phenotypes in Oocytes and Early Embryos

**DOI:** 10.1007/s43032-022-01079-7

**Published:** 2022-10-05

**Authors:** Wenzhu Yu, Shaodi Zhang, Baoli Yin, Chang Dong, Victor Wei Zhang, Cuilian Zhang

**Affiliations:** 1 Department of Reproductive Medicine Center, Henan Provincial People’s Hospital, People’s Hospital of Zhengzhou University, and Henan Provincial People’s Hospital of Henan University, Zhengzhou, Henan China; 2 Henan Joint International Research Laboratory of Reproductive Bioengineering, Zhengzhou, Henan China; 3 AmCare Genomics Lab, Guangzhou, Guangdong China

**Keywords:** Female infertility, *TUBB8*, Oocyte maturation arrest, Mutation, Abnormal fertilization, IVF

## Abstract

Tubulin beta 8 class VIII (TUBB8) is a β-tubulin isotype that is specifically expressed in human oocytes and early embryos. It has been identified as a disease-causing gene in primary female infertility by affecting oocyte maturation arrest. This study investigated the genetic cause of female infertility in five patients from four families. Five women with primary infertility were recruited. Medical-exome sequencing and Sanger sequencing were performed on the patients, and their family members to identify candidate genes that explained infertility. Additionally, the morphology of oocytes and zygotes from the patients and controls were assessed. We observed recurrent oocytes MI arrest, oocytes abnormal fertilization, uncleaved embryos, and embryo transfer failure in the patients. Heterozygous missense variants in *TUBB8*, c.538G > A (p.V180M), c.527C > G (p.S176W), c.124C > G (p.L42V), and c.628A > C (p.I210L), were verified in four unrelated families. This study expanded the mutational spectrum of *TUBB8* by identifying three novel heterozygous missense variants. Screening for *TUBB8* mutation demonstrated the diagnostic utility of female infertility.

## Introduction

Infertility affects approximately 10–15% of couples worldwide, and female infertility accounts for nearly two-thirds of them [1]. Assisted reproductive technologies (ART) such as in vitro fertilization (IVF) and intracytoplasmic sperm injection (ICSI) successfully help many infertile couples to have offspring. However, there are still women experiencing recurrent failures of IVF/ICSI due to oocyte maturation arrest, embryo transfer failure, or embryonic arrest.

Successful human reproduction requires proper oocyte maturation and embryonic development. The meiotic cell cycle of oocyte starts in the neonatal ovary and pauses at prophase I of meiosis until the luteinizing hormone restores meiosis during puberty. Oocyte maturation takes place in the sequence of germinal vesicle (GV) breakdown, spindle assembly, and asymmetric division. This progression completes after extruding the first polar body (PB) and again arrests at the MII stage until fertilization [2]. Any disturbance during these periods can lead to oocyte maturation arresting and result in female infertility [3, 4]. Although several studies have investigated the mechanism of oocyte maturation defects in mice models, the genetic etiology in human remains largely unknown.

Tubulin beta eight class VIII (TUBB8) is a highly conserved primate-specific β-tubulin isotype that is uniquely expressed in human oocytes and early embryos. Spindle assembly plays an important role in both mitosis and meiosis. The spindles consist of dynamic microtubules, which are polymerization by the α/β-tubulin heterodimer [5]. Feng et al. [6] first identified that disease in *TUBB8* gene compromise spindle assembly and then led to oocyte arrest at MI. In the subsequent studies, a phenotype spectrum was discovered, including oocyte maturation arrest at the MI or MII stages, inability for fertilization, recurrent failures of embryo transfer, and embryos that were uncleaved or in early arrest. The inheritance pattern of *TUBB8* and pathogenic mechanisms are also variable. The autosomal dominant form is caused by dominant-negative effects, while autosomal recessive is through haploinsufficiency effects [6, 7]. Previous studies found that mutations in the *TUBB8* gene can explain approximately 30% of women with oocyte maturation arrest [8, 9].

In this study, we reported five female patients from four unrelated families with primary infertility and IVF failure, characterized by a range of oocyte and embryo developmental arrest phenotypes. Medical exome sequencing revealed four heterozygous missense variants in *TUBB8*, of which three variants were novel and one variant was recurrent.

## Materials and Methods

### Human Subjects

**Fig. 1:**
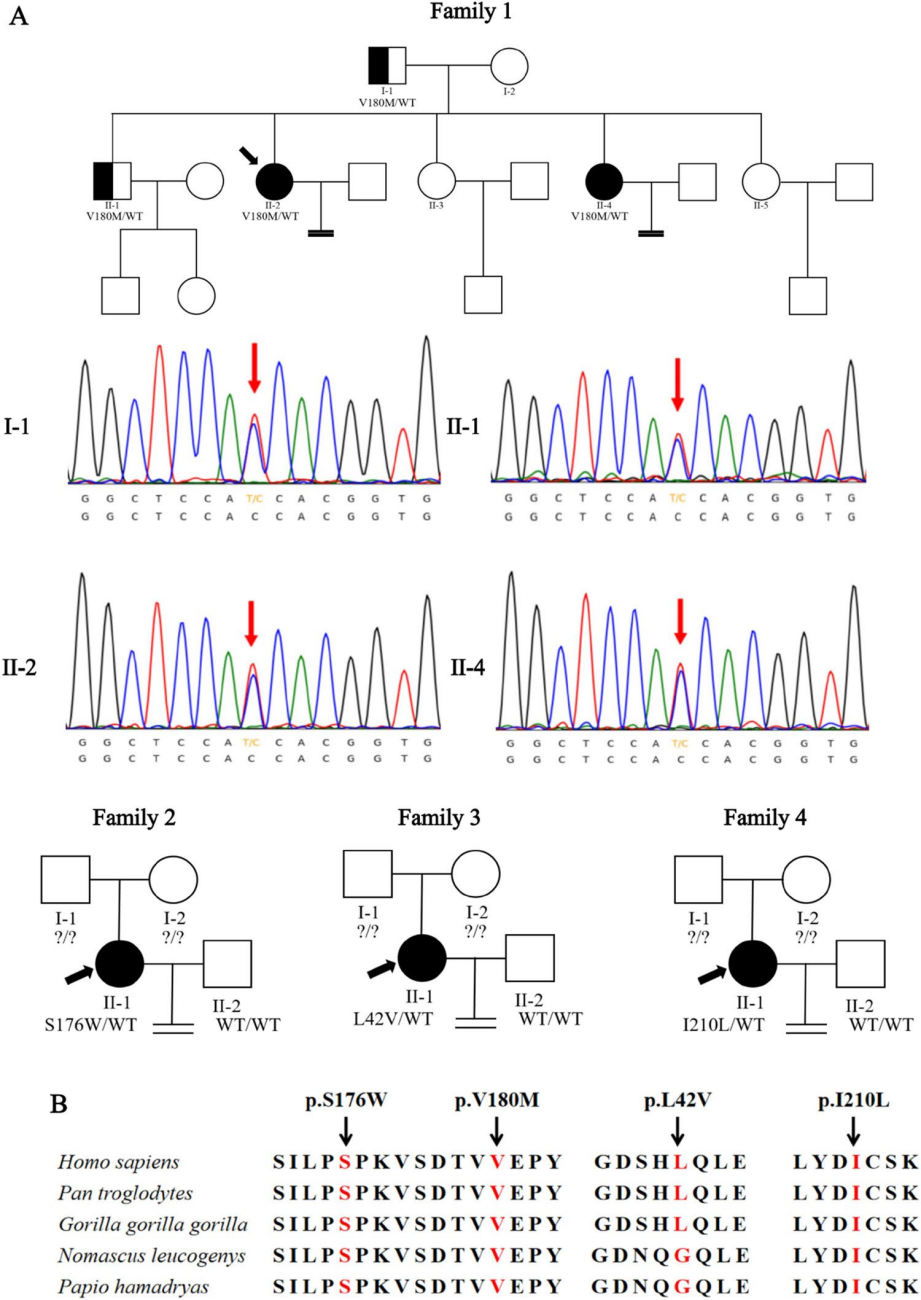
Genetic analysis of *TUBB8* variants. **A** Pedigree of the 4 unrelated families. The black arrow indicated the proband. **B** Multiple alignments of *TUBB8* indicated that p.V180M, p.S176W, and p.I210L are highly conserved among primates

A total of five infertility women due to oocyte maturation arrest, abnormal fertilization, embryonic development disorder, and large polar body oocyte from four families were recruited from the Reproductive Medicine Center, Henan Provincial People Hospital **(**Fig. 1A; Table 1). Informed consent forms were signed by all patients and their family members. Peripheral blood samples were collected from all members for DNA extraction and subsequent sequencing.

**Table 1:** Clinical characteristics of patients and their retrieved oocytes

Case	Family	Age	Duration of infertility (years)	Previous IVF/ICSI cycles	COH protocol	Total oocyte retrieved	GV oocyte	MI oocyte	MII oocyte	Oocytes with abnormal morphology	Normal fertilized oocyte	Abnormal fertilized oocyte	Cleavage embryos
1	Family 1, II-2	29	7	2	1 long + 1 GnRH-ant	15	0	15	0	0	0	0	0
2	Family 1, II-4	24	2	2	2 long	35	0	29	6	0	2	2	0
3	Family 2, II-1	34	7	2	2 long	29	1	22	0	6	0	0	0
4	Family 3, II-1	33	7	4	2 long + 2 μm stimulation	34	6	18	10	0	8	2	8
5	Family 4, II-1	30	4	6	1 long + 4 GnRH-ant + 1 μm stimulation	99	9	17	52	21	19	33	19

*GV* germinal vesicle, *MI* metaphase I, *MII* metaphase II, *IVF* in vitro fertilization, *ICSI* intracytoplasmic sperm injection, *GnRH-ant* GnRH antagonist

### Evaluation of Oocyte and Embryo Phenotype

The morphology and maturity of oocytes or embryos were examined by light microscopy. Both patients followed the controlled ovarian hyperstimulation (COH) protocols, and transvaginal ultrasound was used to monitor the diameters of follicles. After 2–3 dominant follicles reached a diameter of 18 mm, human HCG was injected 36–38 h before oocytes retrieval. Oocytes were retrieved from the patients and underwent IVF/ICSI. At 16–18 h after the insemination, the oocytes were checked for fertilization. The presence of two pronuclei (PN) was considered normal fertilization. The fertilized zygotes were cultured, and morphology was assessed on days 2 and 3 by optical microscope.

### DNA Extraction and Medical Exome Sequencing

A total of 3–5 mL peripheral blood was drawn from the proband and her six family members. The blood samples were sent to the independent laboratory (AmCare Genomics Laboratory, Guangzhou, China) for medical exome sequencing (MES) and data analysis. Genomic DNA was extracted utilizing the Solpure Blood DNA Kit (Magen) following the manufacturer’s instructions. The genomic DNA was fragmented by the Q800R Sonicator (Qsonica) to generate 300–500 bp insert fragments. Custom-designed NimbleGen SeqCap probes (Roche NimbleGen, Madison, WI, USA) were used for in-solution hybridization to enrich target sequences, which included coding exonic regions of approximately 5000 OMIM recorded genes and known pathogenic variants from deep intronic or other non-coding regions. Enriched DNA samples were then sequenced by the Illumina NovaSeq 6000 platform. The average coverage depth was 200 × with coverage greater than 20 × accounting for more than 99% of the exome. Sequenced reads were mapped to the reference human genome version (GRCh37/hg19) by NextGENe. Sequence variants were annotated and included population (gnomAD, 1000 Genomes, dbSNP) and variant databases (Clinvar, HGMD). Variant interpretation followed the American College of Medical Genetics (ACMG) guidelines [10]. Sanger sequencing was performed to confirm the variants identified by MES.

## Results

### Clinical Presentation

A total of 5 infertility women from 4 unrelated families who underwent IVF/ICSI attempts in our center were recruited. They had normal menstrual cycles, basal sex hormone levels, thyroid function, ovary function, and karyotype. All of their husbands were healthy with normal karyotype and normal sperm concentration, motility, and morphology. None of the couples had a family history of infertility (except for Case 2) and no history of exposure to reproductive toxicities. Ultrasound and hysterosalpingography examinations showed that Cases 1, 2, and 3 had obstructed left fallopian tubes, and all patients showed normal development of the uteruses and ovaries.

**Fig. 2:**
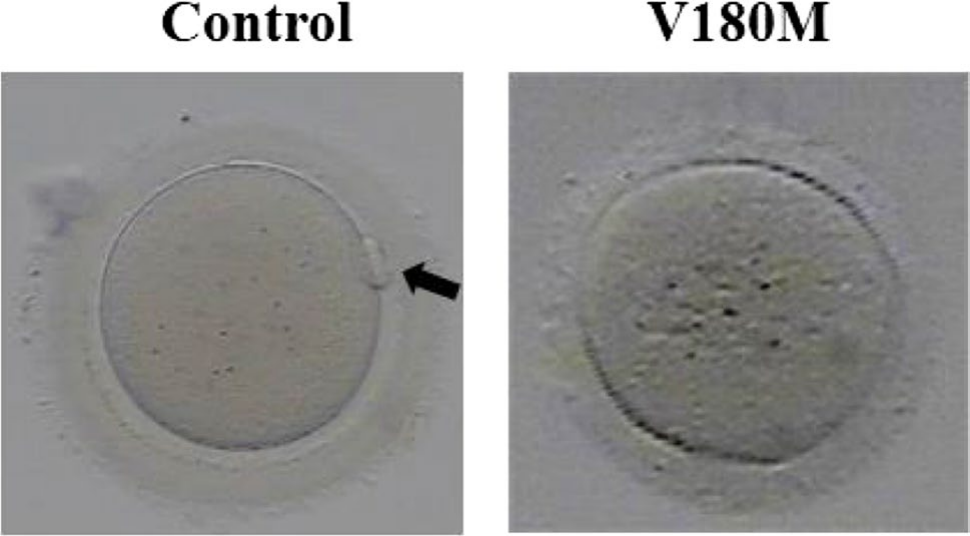
Morphology of oocytes and embryos from patients and control. The morphologies of the MI or MII oocytes were observed by light microscopy. The arrow represents the polar body

Detailed patients’ clinical information including age, the duration of infertility, previous COH protocols, history of IVF/ICSI circles, oocyte maturity and morphology, fertilization, and embryo development outcomes are summarized in Table 1. The five patients showed rather different features of oocytes and embryos. Case 1 (Family 1, II-2) and Case 3 (Family 2, I-1) both showed oocyte maturation arrest; all of their retrieved oocytes were arrested at the MI stage after removing the granulosa cells, and no MII oocyte was found after in vitro maturation cultures (Fig. 2). Both of the patients had no successfully fertilized embryo for transfer; therefore, their IVF/ICSI cycles were canceled. Case 3 also showed oocytes with abnormal morphology, all oocytes without granulosa cells (6/29, 20.7%). Despite Case 2 (Family 1, II-4) coming from the same family with Case 1, her oocyte phenotype was different from her sister. She was able to obtain a small number of MII oocytes (6/35, 17.1%), and two 2PN zygotes and two 0PN2PB zygotes were seen after ICSI. Only the 2PN zygotes were considered normal fertilization, but the embryos were uncleaved after culture, so the circles were canceled. Similarly, Case 4 (Family 3, I-1) was able to obtain mature MII oocytes and had normal fertilized embryos by ICSI, but she failed to be pregnant after embryo transfer. Twenty percent (2/10, 20%) of her oocytes showed abnormal fertilization. Case 5 (Family 4, I-1) received 6 COH circles, and a total of 99 oocytes were obtained. She had a rather high proportion of MII oocytes (52/99, 52.5%), and oocytes with abnormal morphology (21/99, 21.1%). The MII oocytes could be fertilized with a large proportion of abnormal fertilized oocytes (33/52, 63.5%). The abnormal morphologies of oocyte presented the absence of GV, large polar body, multiple polar bodies, and huge oocyte. The abnormal fertilized oocytes mainly showed multiple PN. She tried 4 times of frozen embryo transfer (FET) but failed to conceive. Overall, the 5 patients suffered from infertility lasted range 2–7 years, and they all at least received 2 IVF/ICSI cycles but failed to be pregnant.

### Mutation Analysis

We analyzed the medical exome sequencing data focusing on the known genes related to female infertility. The variant spectra of *TUBB8* in 5 patients are summarized in Table 2. In family 1, a novel heterozygous missense mutation in *TUBB8*, c.538G > A (p.V180M), was found in the proband (II-2), her elderly fertile brother (II-1), and her younger sister (II-4) who also suffered from infertility. The mutation in the three siblings was inherited from their father (I-1). None of the proband’s mother (I-2) and her other two fertile sisters (II-3, II-5) carried the same mutation. In families 2–4, heterozygous mutations c.527C > G (p.S176W), c.124C > G (p.L42V), and c.628A > C (p.I210L) were found in Cases 3, 4, and 5, respectively. Because of a lack of parental information, the three variants were considered as an unknown inheritance pattern (Fig. 1A). We performed in silico prediction for the variants by SIFT and PolyPhen-2; all variants were predicted to be damaging, except p.L42V had a controversial result between two methods (SIFT: damaging; Polyphen-2: benign). We performed additional predictions for p.L42V through PROVEAN (Tolerable) and MutationTaster (Diseasing-causing). As TUBB8 protein is a primate-specific β-tubulin isotype, we also performed multiple alignments across various species, and the result showed that all variants were highly conserved among primates, except for p.L42V (Fig. 1B).

**Table 2:** *TUBB8* variants identified in 4 families

Case	Patients	cDNA change	Protein change	Mutation type	Genotype	Inheritance pattern	gnomAD AF (East Asian)	PPH2	SIFT	ACMG classification
1	Family 1, II-2	c.538G > A	p.V180M	Missense	het	Dominant	0	Possibly damaging	Damaging	VUS
2	Family 1, II-4									
3	Family 2, II-1	c.527C > G	p.S176W	Missense	het	Unknown	0	Probably damaging	Damaging	VUS
4	Family 3, II-1	c.124C > G	p.L42V	Missense	het	Unknown	0.001	Benign	Damaging	VUS
5	Family 4, II-1	c.628A > C	p.I210L	Missense	het	Unknown	0	Probably damaging	Damaging	VUS

*AF* allele frequency, *VUS* variant of uncertain significance, *het* heterozygous, *PPH2* Polyphen-2

## Discussion

Female infertility is a multicausal reproductive disease, which affects 10% of women worldwide [11]. In this study, we reported five patients from four unrelated families with primary infertility, representing oocyte maturation arrest, abnormal fertilization, uncleaved embryos, and embryo transfer failure. Four different missense variants (3 of which are novel) in *TUBB8*, c.538G > A, c.527C > G, c.124C > G, and c.628A > C were identified in each family, respectively.

There were more than 100 unique variants in *TUBB8* that have been reported, most of them were missense, and other mutation types include frameshift, splice site, and small indels [8]. Heterozygous *TUBB8* missense variants that caused oocyte maturation arrest were mostly paternally inherited or de novo through dominant-negative effects, while homozygous variants in consanguineous families and heterozygous variants maternally inherited with incomplete penetrance were also reported [12]. In this study, the variant p.V180M found in Family 1 was identified in Cases 1 and 2, inherited from their father and their fertile brother also carried the variant, while it was not found in the female family members with normal fertility. In this pedigree, the segregation pattern suggested that p.V180M was autosomal dominant inherited. The variant p.V180M was not reported in infertility cases before, while a variant, p.V179M, only one residue apart, was reported in two patients which both showed oocytes arrest at the MI stage [13]. In addition, p.S176L, a variant at the same position with p.S176W but different amino acid change, was reported in two unrelated families and was de novo. The patients who carried p.S176L had no morphologically identifiable MII oocytes, whose phenotypes were consistent with Case 3 who carried p.S176W [6, 14]. The variant p.L42V in Case 4 was previously reported in a patient with embryonic arrest [9]. Case 4 and a former patient who carried p.L42V both could obtain MII oocytes and had normal fertilized embryos, but they either had no usable embryos due to embryonic arrest or suffered embryo transfer failure. Similarly, the variant p.I210L in Case 5 was novel, while a variant at the same position, p.I210V, was reported in a patient with normal fertilization but embryonic arrest [14]. Case 5 had identifiable MII oocytes and normal fertilized embryos, but experienced recurrent embryo transfer failures, which was similar to patient who carried p. I210V. In vivo experiments revealed that p.S176L and p.I210V caused complete loss of the microtubule network and impaired spindle assembly [6, 14]. Moreover, the functional change of p.L42V and p.V180M still require further investigation.

According to the current data, *TUBB8* variants can explain 30% of patients with oocyte maturation arrest [15]. The phenotypic spectrum of oocytes with *TUBB8* deficiency is associated with the defect of spindle assembly. Phenotypes can be variable, even with the same variant in one family. New phenotypes of *TUBB8* variants were successively discovered in recent years, including (1) oocytes arrested at the MI stage, (2) MII oocytes that cannot be fertilized, (3) MII oocytes that can be fertilized but uncleaved, (4) embryos with early arrest, (5) viable embryos but recurrent transfer failure, (6) large polar bodies, and (7) multiple pronuclei in zygotes [6, 12, 14, 15, 16].

The tubulin family has 8 β-tubulin isotypes in human, and different isotypes are distinguished by different coding genes or posttranslational modifications [5]. Beta-tubulin forms heterodimers with α-tubulin and polymerizes into microtubules to carry out cellular functions, including intracellular transport and cell division. Carboxy-terminal tail of β-tubulin isotypes is variable, and these isotypes of β-tubulin are enriched in certain cells or tissue. Variants in *TUBB1*, *TUBB2A*, *TUBB2B*, *TUBB3*, *TUBB4A*, and *TUBB4B* affected neuronal migration, differentiation, and axon maintenance [17]. These variants have been reported concerning tubulinopathies in neurons, resulting in nervous system malformations [18]. On the other hand, *TUBB8* function still remains largely unclear, possibly due to its expression being limited to primate oocytes [19]. Several in vivo studies showed that the missense variants in *TUBB8* caused microtubule disruption and severely or completely impaired spindle assembly [6, 14, 15]. In addition, in vitro studies that expressed mutant TUBB8 in culture cells showed that missense variants did not influence translational efficiency but reduced the yield of de novo assembled heterodimers that could result in tubulin haploinsufficiency [14]. In short, most missense variants in *TUBB8* are likely to influence microtubules by impacting either β-tubulin stability or lateral contacts between protofilaments. Structure analysis of β-tubulin [6, 13] confirmed that p.V179, and p.S176 are located at a longitudinal interface between assembled alpha/beta-tubulin heterodimer and the variants presumably disrupt heterodimer longitudinal interactions and affect microtubule assembly. We speculated that p.V180M, p.S176W, p.L42V, and p.I210L possibly present similar effects.

*TUBB8*-deficiency primary female infertility had a poor prognosis in assisted reproductive treatment. Scientists are attempting to explore therapy for *TUBB8* deficiency in mouse models. Jia et al. [20] found that co-injection of wild-type *TUBB8* cRNA into oocytes that expressed mutant *TUBB8* can successfully improve spindle assembly and produce live offspring. However, centrioles do not participate in mouse fertilization, and the pattern of early embryo cleavage in mice is different from that in human. Therefore, the safety and reliability of gene therapy in human still require further experimental evidence and verification. However, women with infertility caused by *TUBB8* variants were able to conceive through egg donation [21]. *TUBB8* variants have a high proportion of infertile females, and males who carried *TUBB8* variants are fertile. Exome and genome sequencing has not been widely used in infertility clinics. It is possible that the prevalence of *TUBB8* variants may be underestimated. Thus, we suggested that *TUBB8* screening should be performed for women with primary infertility to avoid recurrent IVF failure and to reduce the financial and psychological burden of the patients.

## Conclusion

In summary, we reported 5 women with infertility in 4 families characterized by oocyte maturation arrest, abnormal fertilization, uncleaved embryos, and embryo transfer failure. Three novel variants and one recurrent missense variant were identified in *TUBB8*. Our findings emphasize the importance of screening for TUBB8 variant in infertile patients with oocyte maturation and or embryo development arrest.

## Data Availability

The data in this article are not publicly available because of concerns regarding patient anonymity. Requests to access the data should be directed to the corresponding author.
